# Challenging Diagnosis of Pure Erythroid Leukemia: A Case Report and Literature Review

**DOI:** 10.3390/hematolrep14010007

**Published:** 2022-03-19

**Authors:** Shingo Sato, Masayuki Kobayashi, Ken Suzaki, Ittoku Nanke, Nobuharu Kosugi

**Affiliations:** 1 Department of Internal Medicine, Tokyo Metropolitan Bokutoh Hospital, Tokyo 130-8575, Japan; satoshin.0x0.7@gmail.com (S.S.); reilaxon@gmail.com (I.N.); 2 Department of Hematology, Tokyo Metropolitan Bokutoh Hospital, Tokyo 130-8575, Japan; ken.suzaki59@gmail.com (K.S.); nobuharu_kosugi@tmhp.jp (N.K.)

**Keywords:** pure erythroid leukemia, PEL, acute myeloid leukemia, AML, epithelial tumor

## Abstract

Pure erythroid leukemia (PEL) is an extremely rare type of acute myeloid leukemia (AML), accounting for fewer than 1% of all AML cases. A 72-year-old man presented with severe fatigue. His bone marrow aspiration contained myeloperoxidase negative abnormal cells that were aggregating and depicting epithelial adhesion, suggesting the possibility of solid tumor metastasis. His general condition deteriorated during medical diagnosis, and he died soon after starting chemotherapy. PEL appeared to be the definitive diagnosis after evaluating the histopathological findings, which were obtained after his death. With atypical morphological features, immunophenotypic and karyotypic approaches must be integrated for PEL assessment.

## 1. Introduction

Pure erythroid leukemia (PEL) is a rare and aggressive form of acute myeloid leukemia (AML). According to the 2016 World Health Organization (WHO) classification, PEL is the only type of acute erythroid leukemia that consists of >80% of immature erythroid precursors with ≥30% proerythroblasts among all nucleated bone marrow cells [1]. PEL accounts for <1% of all AML cases, and it has a very poor prognosis; the median survival time is about three months [2]. The cells of PEL are typically large with central round nuclei and irregular nucleoli and have basophilic cytoplasm with vacuoles. There is no established therapy for PEL, and induction chemotherapy and hypomethylating agents are the current treatment options [3]. A limited number of reports suggests that PEL may evolve from pre-existing myelodysplastic syndrome (MDS), and prior chemoradiotherapy exposure could also affect its incidence [4]. Since PEL is extremely rare and its diagnosis can sometimes be challenging, further information on its clinical course, diagnosis, and pathophysiology is crucial to develop novel treatment options and appropriate palliative care. Therefore, our aim is to contribute towards its diagnosis and, moreover, shed light on the importance of a differential diagnosis for PEL from various other AML. Here, we report a case of de novo PEL that was challenging to diagnose. This study was approved by the Institutional Review Board and was conducted according to the principles of the Declaration of Helsinki.

## 2. Case Report

A 72-year-old man was referred to our hospital with complaints of lumbago and severe fatigue. The patient denied weight loss, melena, night sweats, chills, or fever. On physical examination, conjunctival pallor was observed, with no enlarged lymph node detectable from the body surface. Laboratory testing showed anemia (Hb 7.6 g/dL), severely increased lactate dehydrogenase level (22,003 U/L), ferritin (17,040 ng/mL), and C-reactive protein (30.95 mg/dL). A peripheral blood smear showed a decrease in red blood cells; however, the morphology was normal with no blast cells. Contrast-enhanced computed tomography (CT) revealed a low-density area around the Th12 vertebral body and osteolytic changes in the sacrum and ilium; however, no lymphadenopathy was found. Head magnetic resonance imaging revealed several acute cerebral microinfarctions in the left cerebral hemisphere.

**Figure 1:**
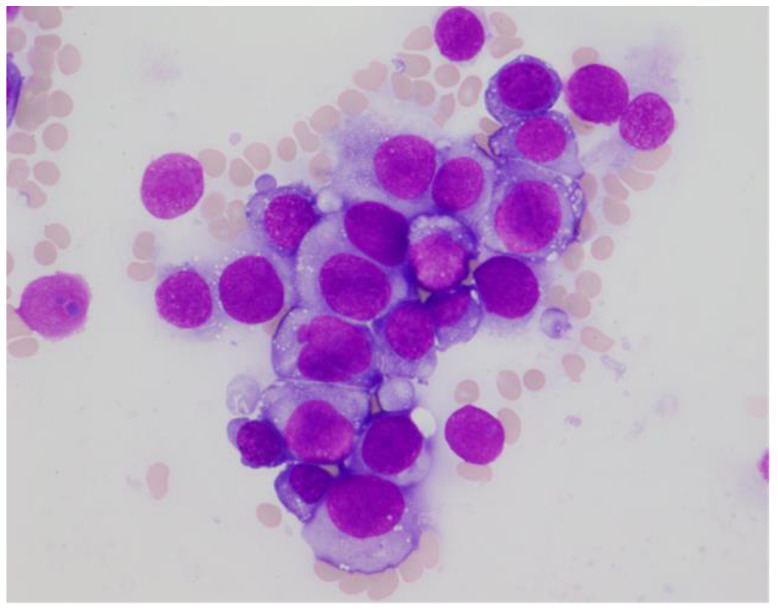
Bone marrow smears on admission (May-Giemsa staining, ×400). A majority of the nucleated cells were medium- to large-sized abnormal cells. Abnormal cells had condensed chromatinated nuclei and distinct nucleoli, with basophilic cytoplasm with cytoplasmic blebs and vacuoles.

Based on stroke-like episode with extremely high lactate dehydrogenase, intravascular lymphoma (IVL) was considered as a differential diagnosis at this point. Therefore, we conducted a random skin biopsy to evaluate IVL; however, the result was negative. Initial bone marrow aspiration was a “dry tap”; therefore, core biopsy was performed. On the second aspiration after the biopsy, a small amount of bone marrow fluid was noted. A smear of the bone marrow fluid showed slight hypocellularity, with 64% of myeloperoxidase (MPO) negative abnormal cells. The abnormal cells were medium to large sized with condensed chromatinated nuclei and distinct nucleoli, and had basophilic cytoplasm with cytoplasmic blebs and vacuoles (Figure 1).

**Figure 2:**
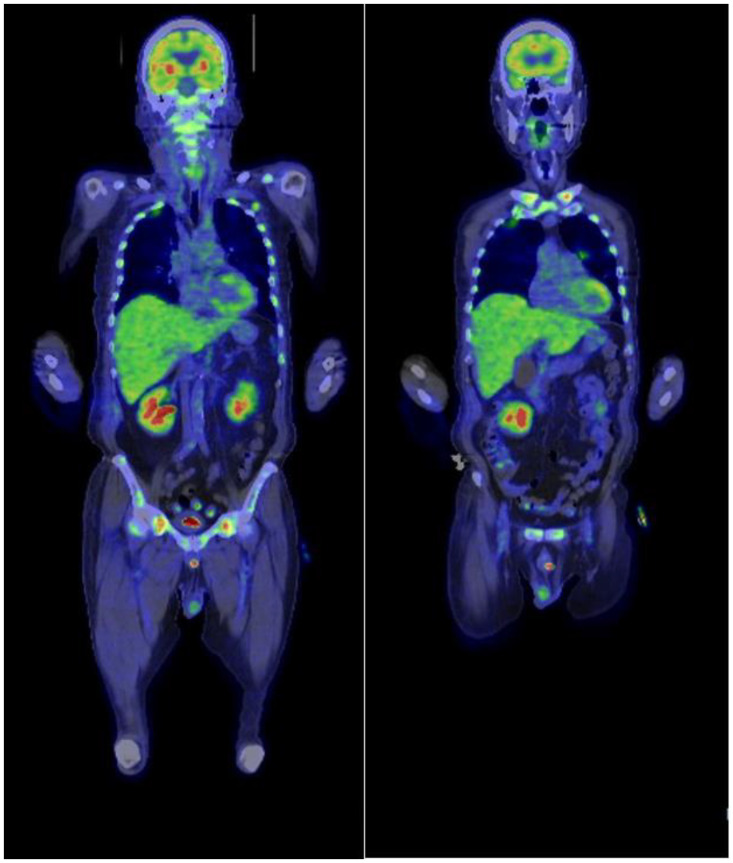
A lesion exhibiting high 18-FDG uptake (SUVmax, >4) was detected in the bones (clavicle, humerus, spine, rib, pelvis, and femur) and spleen. 18-FDG, 2-deoxy-2-(fluorine-18)-fluoro-D-glucose; PET, positron emission tomography; SUVmax, maximum standard uptake value.

PEL was one of the differential diagnoses at this point; however, the abnormal cells also showed aggregation and cell–cell adhesion, suggesting the possibility of epithelial cancer metastasis. There were no blast cells or abnormal cells found in peripheral blood, and osteolytic changes in the sacrum and ilium further supported this possibility. Therefore, we searched for a primary cancer while we waited for the result of the bone marrow biopsy and its immunohistochemistry. No remarkable lesions were found in the upper and lower gastrointestinal endoscopy. Fluorodeoxyglucose-positron emission tomography (FDG-PET) demonstrated a high level of accumulation in the femur, sacrum and ilium (Figure 2).

It also revealed moderate accumulation in bones of the whole body and the spleen, and slight accumulation in the left local lung field. The results of the flow cytometry analysis of the bone marrow aspirates were available on day 8. No significant results were observed on performing the 7-amino actinomycin D cell viability flow cytometry analysis targeting lymphoma. Analyses with CD45/side scatter gating demonstrated that abnormal cells were partially positive for CD4 (35.3%), CD13 (48.8%), CD33 (61.6%), CD71 (43.7%), glycophorin A (29.1%), and HLA-DR (55.3%), whereas other myeloid and lymphoid markers were negative. However, the results were unremarkable due to the lack of samples, as there were merely 132 cells available for the count. Therefore, we additionally performed a CT-guided biopsy of the sacrum. Just after the CT-guided biopsy, his general condition deteriorated. His body temperature increased up to 38 degrees Celsius, and lactate dehydrogenase and total bilirubin levels increased drastically to 39,026 U/L and 9.73 mg/dL.

**Figure 3:**
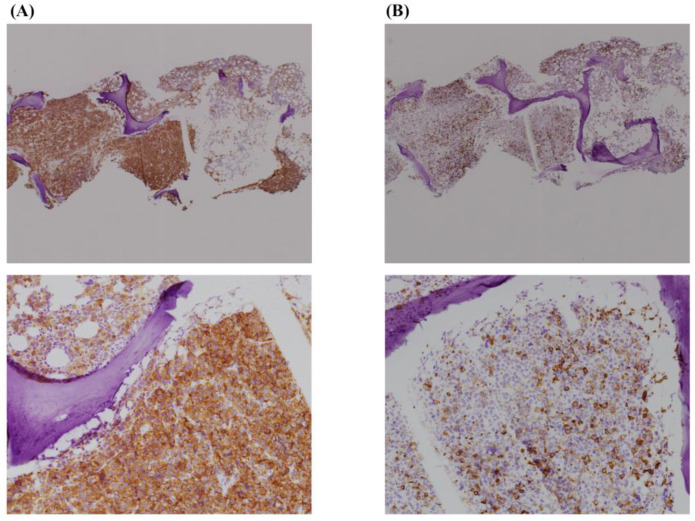
Immunohistochemical staining of bone marrow biopsy. (**A**) Nearly all the nucleated cells were positive for CD71. (×40, ×100) (**B**) Cells were weakly positive for Glycophorin A (×40, ×100).

Since none of the inspections revealed any primary solid cancer and the immunostaining was yet to be reported, induction chemotherapy was performed with possible diagnosis of PEL, which comprised daunorubicin and cytarabine. However, the treatment was ineffective, and the patient died of multiple organ failure three days after initiating the treatment. After his death, the result of the biopsy was reported; nearly all the nucleated cells were positive for CD71 and weakly positive (<50%) for Glycophorin A (Figure 3). In conjunction with morphological characteristics, a definitive diagnosis of PEL was made.

## 3. Discussions

We herein report a rare case of de novo PEL mimicking an epithelial tumor metastasis. In our case, with atypical morphological features and laboratory data, immunophenotypic and karyotypic approaches were the keys for the diagnosis of PEL, which underscored the importance of comprehensive assessment of PEL. To meet the diagnostic criteria for PEL, ≥20 percent of total nucleated cells should be blasts (including pronormoblasts), and cells of erythroid lineage should account for >80 percent of bone marrow cellularity [1]. The erythroblasts do not express markers of myeloid lineage and do not stain with MPO. They react with antibodies to glycophorin A and may express CD117. Ever since PEL was first described by Di Guglielmo in 1928 [5], PEL remains a rare entity among all AML diseases. Owing to its low incidence, making a precise diagnosis of PEL can sometimes be challenging. In our case, the bone marrow heavily comprised abnormal cells during the initial bone marrow aspiration; therefore, we could not collect enough samples for flow cytometry and had to wait for the histopathological analysis. Moreover, the abnormal cells showed aggregation and cell–cell adhesion, suggesting the possibility of epithelial cancer metastasis, which further delayed the diagnosis. We suggest that flow cytometric immunophenotyping is the most important examination for early diagnosis. Therefore, even if it is difficult to obtain bone marrow aspirates, bone marrow aspiration from multiple sites may be useful.

We searched PubMed for articles published since 2010, with the keywords “pure erythroid leukemia.” We found 64 articles in total, 13 of which were case reports on PEL developed in adults (Table 1). Of the 13 cases, 9 (82%) involved pancytopenia on first admission, 2 involved bicytopenia, whereas no laboratory data were available for the other 2 cases. Proerythroblasts can be found in peripheral blood in some cases. In our case, white blood cells (WBC) were within the normal range and platelets were found to be slightly decreased; however, it is debatable whether the laboratory data should have been interpreted as bicytopenia on admission. As Wong E et al. reported [4], PEL usually evolves from pre-existing MDS or due to a medical history of chemoradiotherapy. Of the 13 patients with PEL, excluding 2 cases for which medical history were not available, 7 (64%) received either chemotherapy or radiotherapy before the diagnosis of PEL, and only 2 cases (18%) had no medical history. In our case, the patient did not receive chemotherapy or radiotherapy before admission and his medical history only included hypertension and old cerebral infarction, which was atypical for PEL. 

In the smear of a few bone marrow fluids, abnormal cells that could be considered as erythrocytic precursors represented only 64% of all nuclear cells, which did not meet the diagnostic criteria for PEL. Most importantly, the abnormal cells tended to aggregate and showed cell–cell adhesion in the specimen. Considering the above reasons, it was feasible to assume that a primary cancer was present, and the bone marrow lesion was a metastasis; consistent with the fact that there were no blast cells found in peripheral blood and osteolytic changes were detected in the sacrum and ilium. In the end, the bone biopsy revealed that nearly all nuclear cells were CD71 positive and partially positive (<50%) for Glycophorin A. Therefore, in conjunction with morphological characteristics, PEL was established as the final diagnosis. 

Reinig et al. reported the clinicopathologic and cytogenetic characteristics of 15 de novo PEL cases diagnosed at the Mayo Clinic [19]. Laboratory data of 14 patients were available, of which, the data for 7 patients revealed pancytopenia and the data for the other 7 revealed bicytopenia, which is consistent with the data shown in Table 1. Circulating proerythroblasts were found in 10 cases (67%). The median overall survival was 1.4 months. Interestingly, from a pathological perspective, proerythroblast counts of the bone marrow aspirate did not correspond to that of the bone marrow core biopsy in 8 of the 15 cases. In inadequate bone marrow aspirate specimens, proerythroblast counts are sometimes underestimated to not meet the WHO criteria for PEL, which could lead to a misdiagnosis. In our case, we managed to obtain very few bone marrow fluids after the core biopsy, through forced aspiration. Therefore, it was understandable that proerythroblast counts were underestimated in the initial smear, which made it difficult to precisely diagnose it earlier. Reinig et al. has also mentioned that proerythroblasts might appear cohesive, mimicking a metastatic tumor when they uniformly form large and expansive foci [19]. In our case, the aggregation and cell–cell adhesion made it difficult to distinguish between PEL and epithelial cancer metastasis. There are no reports referring to the morphological similarities between them, which made a precise diagnosis challenging in the 13 case reports as shown in Table 1. Since the results of immunophenotypic inspections take several days to weeks, further studies and case reports are required to better understand the morphological features of PEL, which will effectively help us distinguish PEL from epithelial cancer metastasis. 

## 4. Conclusions

In conclusion, we encountered an extremely rare de novo PEL case in which morphological features and inadequate bone marrow aspirate, along with laboratory data and CT findings made the precise diagnosis of PEL even more challenging and particularly difficult to distinguish from epithelial cancer metastasis. Even if immature erythroid precursor counts or proerythroblast counts of the bone marrow aspirate do not fulfill the criteria for PEL, PEL should not be excluded because there are many cases where bone marrow core biopsy has revealed sufficient erythroid lineage to be diagnosed as PEL. As in our case, morphological features and laboratory data could be sometimes atypical for PEL; therefore, immunophenotypic and karyotypic approaches must be integrated to make a comprehensive assessment of PEL, even if there is a slight possibility of PEL.

## Figures and Tables

**Table 1:** Reported case of PEL developed in adults since 2010.

No	Age	Sex	Laboratory Data	Underlying Disease	Prior Treatment	Reference
1	44	F	pancytopenia	none	-	Linnik et al. 2019 [6]
2	69	F	pancytopenia	polymyositis	azathioprine	Imataki et al. 2018 [7]
3	69	M	bicytopenia	N/A	N/A	Caldwell et al. 2019 [8]
4	64	F	bicytopenia	breast cancer	chemo and radio	Niscola et al. 2013 [9]
5	65	M	N/A	plasma cell myeloma	chemo and SCT	Thakral et al. 2017 [10]
6	65	M	pancytopenia	N/A	N/A	Gajendra et al. 2019 [11]
7	68	M	N/A	polycythemia vera	chemo	D. Ware et al. 2018 [12]
8	48	M	pancytopenia	none	-	Aljabry et al. 2015 [13]
9	68	M	pancytopenia	HIV	antiretroviral drug	J. Oberley et al. 2014 [14]
10	66	M	pancytopenia	hypopharyngeal carcinoma	chemo and radio	Funakosi et al. 2011 [15]
11	75	M	pancytopenia	CLL	chemo	Sadrzadeh et al. 2012 [16]
12	42	M	pancytopenia	ALL	chemo	Gupta et al. 2014 [17]
13	69	M	pancytopenia	FL, prostate cancer	chemo and radio	Roquiz et al. 2014 [18]

F; female, M; male, N/A; Not available, SCT; stem cell transplant, HIV; human immunodeficiency virus, CLL; chronic lymphocytic leukemia, ALL; acute lymphocytic leukemia, FL; follicular lymphoma, chemo; chemotherapy, radio; radiotherapy, SCT; stem cell transplant.

## Data Availability

Not applicable.
